# Surprising complexity of the ancestral apoptosis network

**DOI:** 10.1186/gb-2007-8-10-r226

**Published:** 2007-10-24

**Authors:** Christian M Zmasek, Qing Zhang, Yuzhen Ye, Adam Godzik

**Affiliations:** 1 Burnham Institute for Medical Research, North Torrey Pines Road, La Jolla, CA 92037, USA; 2 School of Informatics, Indiana University, E.10th Street, Bloomington, IN 47408, USA; 3 Skaggs School of Pharmacy and Pharmaceutical Sciences, University of California San Diego, Gilman Drive, La Jolla, CA 92093, USA

## Abstract

**Background:**

Apoptosis, one of the main types of programmed cell death, is regulated and performed by a complex protein network. Studies in model organisms, mostly in the nematode *Caenorhabditis elegans*, identified a relatively simple apoptotic network consisting of only a few proteins. However, analysis of several recently sequenced invertebrate genomes, ranging from the cnidarian sea anemone *Nematostella vectensis*, representing one of the morphologically simplest metazoans, to the deuterostomes sea urchin and amphioxus, contradicts the current paradigm of a simple ancestral network that expanded in vertebrates.

**Results:**

Here we show that the apoptosome-forming CED-4/Apaf-1 protein, present in single copy in vertebrate, nematode, and insect genomes, had multiple paralogs in the cnidarian-bilaterian ancestor. Different members of this ancestral Apaf-1 family led to the extant proteins in nematodes/insects and in deuterostomes, explaining significant functional differences between proteins that until now were believed to be orthologous. Similarly, the evolution of the Bcl-2 and caspase protein families appears surprisingly complex and apparently included significant gene loss in nematodes and insects and expansions in deuterostomes.

**Conclusion:**

The emerging picture of the evolution of the apoptosis network is one of a succession of lineage-specific expansions and losses, which combined with the limited number of 'apoptotic' protein families, resulted in apparent similarities between networks in different organisms that mask an underlying complex evolutionary history. Similar results are beginning to surface for other regulatory networks, contradicting the intuitive notion that regulatory networks evolved in a linear way, from simple to complex.

## Abstract 2

A comparative genomics approach revealed that the genes for several components of the apoptosis network with single copies in vertebrates have multiple paralogs in cnidarian-bilaterian ancestors, suggesting a complex evolutionary history for this network.

## Background

**Figure 1:**
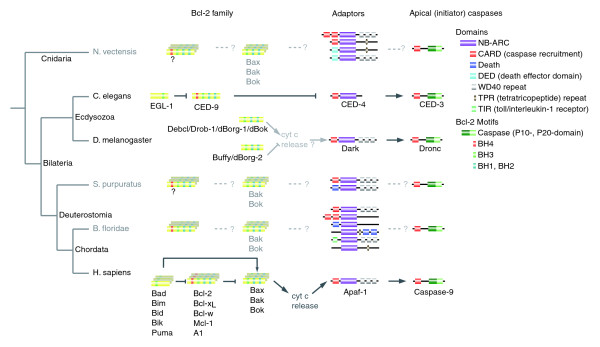
Overview of the initiation of the intrinsic apoptosis pathway. Annotations and domain compositions for *N. vectensis *(sea anemone), *S. purpuratus *(sea urchin), and *B. floridae *(amphioxus) are based on analyses performed in this work, whereas data for *C. elegans*, *D. melanogaster*, and *Homo sapiens *are based on literature [1,2,11]. (Protein and domain lengths are not to scale. In our analysis we noticed a few additional, spurious domains in some CED4/Apaf-1 family members; these are not shown in this diagram.) On the left side, a current view of metazoan phylogeny is shown [13].

Apoptosis is the best-known type of programmed cell death and plays important roles in development and homeostasis as well as in the pathogenesis of many diseases [1,2]. Classical studies on apoptosis in the nematode *Caenorhabditis elegans *identified at first three (CED-3, CED-4, CED-9) and later a fourth protein (EGL-1) to be directly involved in apoptosis [3]. Homologs of the first three proteins were found in genomes of all animals and for all systems studied were shown to be involved in apoptosis (although, the evidence that CED-9 homologs regulate apoptosis in *Drosophila melanogaster *is only indirect) [4,5]. Therefore, they logically were assumed to form the core of the apoptosis network (for an overview, see Figure 1) [1].

Compared to *C. elegans*, the vertebrate apoptosis network is extensive, both in the number and in the size of the protein families involved. While *C. elegans *has one homolog of each (CED-3, CED-4, and CED-9), human has 12 CED-3 (caspase) homologs and 13 CED-9 homologs (Bcl-2-like proteins containing multiple BH motifs) as well as a number of highly divergent proteins that play an analogous role to the EGL-1 protein (BH3 motif only) (three additional caspase related genes, for which confirmation for a role in apoptosis is absent, have been found in *C. elegans*) [6-8]. All mammals, as well as birds, amphibians, and, to a lesser degree, fish, show somewhat similar expansions of these families [9]. The CED-4/Apaf-1 family is an exception, being the only protein from the core of the apoptosis network that was not duplicated in any of the genomes studied until recently. Therefore, it was logical to expect that the role of this protein is indeed central and unique and that all homologs studied to date represent one-to-one orthologs that have evolved by speciation events only. Such one-to-one orthologs usually tend to display a high level of functional similarity and could be effectively used as functional models of each other [10]. In this context, it was somewhat puzzling that an increasing body of experimental evidence suggested fundamental functional differences between *C. elegans *CED-4 and *Drosophila *Dark and their homologs in other species. In vertebrates, cytochrome *c *binds to Apaf-1 to trigger assembly of the apoptosome [6], which in turn leads to caspase activation. In contrast, no cytochrome *c *binding has been recognized for *C. elegans *CED-4 and remains controversial for *Drosophila *Dark [5,11].

With the recent completion of three marine invertebrate genomes, namely two from *Deuterostomia *(the sea urchin *Strongylocentrotus purpuratus *and the amphioxus *Branchiostoma floridae*; unpublished; see Materials and methods) and one from *Cnidaria *(the sea anemone *Nematostella vectensis*), we are now able to obtain a more complete picture of how the complex vertebrate apoptosis network might have evolved and how representative the simple networks seen in insects and nematodes are of the systems present in other invertebrate animals [12-15].

## Results

The assumption that the major expansion of the apoptotic networks is specific to vertebrates was challenged by the results of several studies of individual protein families [16], such as the presence of multiple Bax- and Bak-like sequences in the cnidarian *Hydra magnipapillata *[17], but the assumption was finally laid to rest by the analysis of the recently sequenced sea urchin genome, which showed that many groups of proteins related to apoptosis underwent major expansion in this organism compared not only to *C. elegans*, but also to vertebrates (Table 1) [12,18]. Some groups of apoptosis-related proteins have ten times more members in sea urchin than in corresponding families in vertebrates! The recently sequenced amphioxus genome shows similar expansion. However, the origin of the major expansion of the apoptosis network was moved back in time even further by the analysis of the genome of the morphologically simplest metazoan sequenced to date, the cnidarian *N. vectensis*. Cnidarians are the sister-group of the bilaterian metazoans, with both groups splitting about 650-1,000 million years ago [14]. Yet, both the size of most families of apoptosis domains and proteins as well as the presence of many vertebrate-like subfamilies strongly suggest that the cnidarian-bilaterian ancestor had an apoptosis network comparable in its complexity to that of vertebrates and that the apparent simplicity seen in insects and nematodes is a result of massive gene loss.

**Table 1:** Core apoptosis domains in several completed animal genomes

Classification	Species	NB-ARC domain	Bcl-2 (multi-motif)	Caspase	CARD domain	Death domain (DD)	Death effector domain (DED)
Vertebrata	*H. sapiens *(human)	1 (1)	17 (12)	11 (11)	23 (22)	31 (29)	8 (8)
	*M. musculus *(mouse)	1 (1)	15 (11)	9 (9)	23 (21)	28 (25)	6 (6)
	*C. familiaris *(dog)	1 (1)	14 (10)	14 (14)	20 (19)	37 (33)	5 (5)
	*G. gallus *(chicken)	1 (1)	13 (7)	13 (13)	13 (12)	30 (24)	6 (6)
	*X. tropicalis *(western clawed frog)	1 (1)	14 (11)	13 (13)	28 (28)	31 (28)	5 (5)
	*B. rerio *(zebrafish)	1 (1)	16 (13)	21 (21)	30 (28)	35 (33)	5 (5)
	*F. rubripes *(Japanese pufferfish)	1 (1)	15 (12)	13 (13)	15 (14)	32 (28)	6 (6)
	*T. nigroviridis *(green pufferfish)	1 (1)	13 (11)	14 (14)	14 (12)	33 (30)	5 (4)
Cephalochordata	*B. floridae *(amphioxus)	16 (16)	7 (7)	53 (53)	84 (84)	139 (136)	57 (57)
Urochordata	*C. intestinalis *(sea squirt)	0*	1 (1)	11 (11)	2 (2)	5 (4)	2 (2)
Echinodermata	*S. purpuratus *(purple sea urchin)	5 (5)	8 (8)	42 (42)	12 (10)	87 (82)	3 (3)
Ecdysozoa	*D. melanogaster *(fruit fly)	1 (1)	2 (2)	7 (7)	1 (0)	5 (5)	0
	*C. elegans*	1 (1)	1 (1)	5 (5)	1 (1)	2 (2)	0
Cnidaria	*N. vectensis *(starlet sea anemone)	4 (4)	11 (11)	10 (10)	8 (8)	5 (5)	9 (9)

The total numbers of full-length protein sequence matches to the corresponding human sequences are shown; the number of hits confirmed by Pfam and CD-Search under default thresholds displayed in parentheses (see Materials and methods). We have to stress that the number of proteins in all recently sequenced genomes is approximate because of the diversity of domain sequences and experimental verification of only limited numbers of gene predictions. Therefore, exact counts of the members of these families strongly depend on significance thresholds for gene predictions and specific homology-recognition tools used in the analysis. *We were unable to detect an NB-ARC domain in *C. intestinalis*, probably due to sequence/assembly problems in this genome.

**Figure 2:**
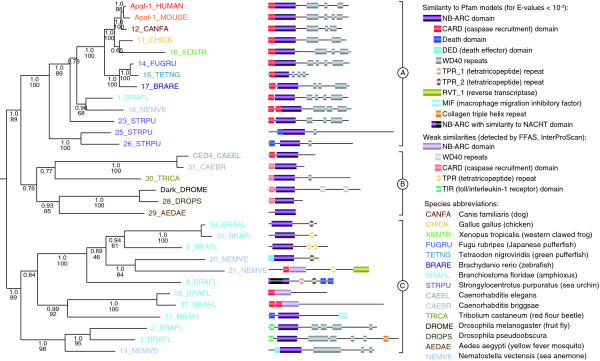
Phylogeny and domain organization of CED-4/Apaf-1 homologs. This phylogeny was calculated using a Bayesian approach (MrBayes) based on a MAFFT alignment of the NB-ARC domains. Posterior probability values are shown for each branch (top numbers). Bootstrap support values for branches that are supported by a minimal evolution method (FastME) based on a PROBCONS alignment are also shown (bottom numbers; for detailed information, see Materials and methods). Furthermore, phylogenies based on full-length alignments of the subset of all Apaf-1 homologs exhibiting a CARD-NB-ARC-WD40 domain composition (all vertebrate sequences, 1_BRAFL, 18_NEMVE, and Dark_DROME) as well as 28_DROPS, CED4_CAAEL, and 31_CAEBR showed precisely the same picture: a clade of vertebrate, amphioxus, and *Nematostella *sequences under exclusion of insect and nematode sequences. For a detailed list of protein sequences see Additional data file 2. For clarity, sequences from *S. purpuratus *(2), and *B. floridae *(6), which appear to be redundant and/or results of erroneous assemblies, are not included in this figure; however, their inclusion/exclusion does not change the quality/interpretation of this phylogeny. All sequences are from complete genomes, except the individual sequences from *Aedes aegypti*, *Caenorhabditis briggsae*, *Drosophila pseudoobscura*, and *Tribolium castaneum*.

Detailed phylogenetic analysis of the central, nucleotide-binding domain of the CED-4/Apaf-1 family shows a somewhat unexpected picture (Figure 2). This domain, classified as NB-ARC (for nucleotide-binding adaptor shared by Apaf-1, R proteins, and CED-4) is a subfamily member of the very large family of AAA+ ATPases [19-21]. NB-ARC is distantly homologous to, but distinctively different from, other nucleotide-binding domains, such as the NACHT domain present in families of proteins involved in immunity [22]. A well-supported subtree, containing human Apaf-1 and its vertebrate one-to-one orthologs, also contains amphioxus, sea urchin, and *Nematostella *sequences, but none from nematodes or insects (subtree A in Figure 2). Evidently, nematode/insect homologs from this subfamily have been lost, thus leaving nematodes/insects without orthologs of human Apaf-1. Nematode and insect proteins form their own subtree (B), diverging from the Apaf-1 branch in a way suggesting that these proteins belong to a separate subtype that was already present at the cnidarian-bilaterian split. Interestingly, several *Nematostella *and amphioxus homologs form additional subfamilies (C), which were lost in both nematodes/insects and vertebrates, indicating an evolutionary history for Apaf-1 predecessors rich in gene duplications and gene losses.

The presence of numerous CED-4/Apaf-1 homologs in the common ancestor of Bilateria and Cnidaria suggests that initially there might have been several mechanisms to activate the intrinsic apoptosis pathways and/or several downstream pathways activated by similar signals and that the mechanism of human Apaf-1 and its vertebrate orthologs presents only one of several possibilities. This also explains why the biochemical/structural mechanism of *C. elegans *CED-4 and *Drosophila *Dark can be significantly different from human Apaf-1 [11].

The functional variations among different branches of the Apaf-1 family are illustrated by their different domain organizations. Human Apaf-1 and its *Nematostella*, amphioxus, and sea urchin homologs exhibit the same or similar domain organization (CARD [two for *Nematostella*]-NB-ARC-WD40 repeats). Nematode and most, but not all, insect sequences seem to lack WD40 repeats [23], suggesting that the loss of the receptor domain of CED-4 is a (relatively) recent event, specific to nematode/insect Apaf-1 homologs. The expanded repertoire of CED-4/Apaf-1 homologs in sea urchin, amphioxus, and *Nematostella *contains proteins with novel domain combinations. This includes replacement of the single CARD domain at the amino terminus with pairs of CARD domains (*Nematostella *and amphioxus), death domains (amphioxus and, as previously described in [18], sea urchin), death effector domains (*Nematostella*), and TIR domains (amphioxus), all of which function as protein-protein interaction facilitators [24]. At the carboxyl terminus, the WD40 repeats are occasionally missing, replaced by TPR repeats [25], or supplemented by double death domain repeats. Therefore, it seems that functional differences among CED-4/Apaf-1 homologs could include both the sensing mechanism (carboxy-terminal receptor domains) and the downstream recruitment function (amino-terminal protein-protein interaction domains). While we can only speculate on how such a rich set of domain combinations (as seen in amphioxus) came to be, a correlation between domain versatility and abundance has been observed [26]. Interestingly, the TIR-NB-ARC domain architecture, present in one of the amphioxus proteins, resembles plant disease-resistant (R) genes involved in a process called hypersensitive response [27], which bears some similarity to apoptosis in animals [28], suggesting possibly even more distant evolutionary connections.

**Figure 3:**
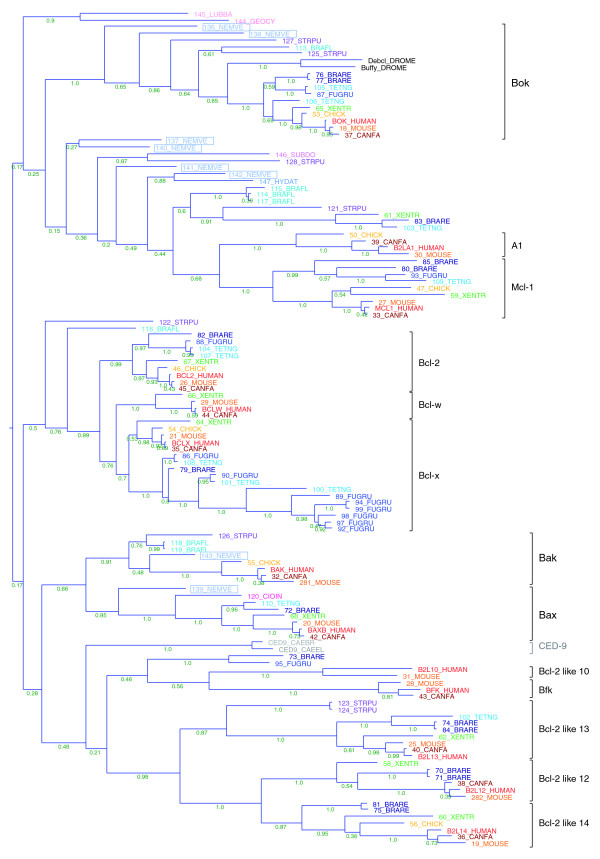
Phylogeny of the multi-motif Bcl-2 family. This phylogeny was calculated using a Bayesian approach (MrBayes) based on a MAFFT alignment of Bcl-2 domains. Posterior probability values are shown for each branch (for detailed information, see Materials and methods). Species abbreviations: BRAFL, *Branchiostoma floridae *(amphioxus); BRARE, *Brachydanio rerio *(zebrafish); CAEBR, *Caenorhabditis briggsae*; CAEEL, *Caenorhabditis elegans*; CANFA, *Canis familiaris *(dog); CHICK, *Gallus gallus *(chicken); CIOIN, *Ciona intestinalis *(sea squirt); DROME, *Drosophila melanogaster *(fruit fly); FUGRU, *Fugu rubripes *(Japanese pufferfish); GEOCY, *Geodia cydonium *(sponge); HYDAT, *Hydra attenuata*; LUBBA, *Lubomirskia baicalensis *(freshwater sponge); NEMVE, *Nematostella vectensis *(starlet sea anemone); STRPU, *Strongylocentrotus purpuratus *(purple sea urchin); SUBDO, *Suberites domuncula *(sponge); TETNG, *Tetraodon nigroviridi*s (green pufferfish); and XENTR, *Xenopus tropicalis *(western clawed frog). For a detailed list of protein sequences see Additional data file 3. All sequences are from complete genomes except the individual sequences from *C. briggsae*, *G. cydonium*, *H. attenuata*, *L. baicalensis*, and *S. domuncula*.

The evolutionary histories of two other protein families playing central roles in apoptosis, Bcl-2 [2] and caspases [29], show very similar pictures (Figure 1): members of major subfamilies were most likely present in the early ancestors but were subsequently lost in nematodes and insects [18,30]. Phylogenetic analysis of multi-motif Bcl-2 family members shows that the Bax, Bak, and Bok groups of proapoptotic Bcl-2 homologs appear to be ancient and that each has at least one well-supported ortholog in *Nematostella *(Figure 3). The many other *Nematostella *Bcl-2 family members are hard to assign to a specific subtype, although one of them (140_NEMVE) contains a putative BH4 motif that makes it similar to the Bcl-2/Bcl-x type. Similarly, Bak and Bok appear to have representatives in sea urchin and amphioxus, both of which also contain a multitude of additional Bcl-2 family genes, which are difficult to consign to a subtype. This is in sharp contrast to the model organisms *D. melanogaster*, which contains only two Bcl-2 family genes belonging to the Bok group (Debcl and Buffy), and *C. elegans*, which has one (CED-9), which is difficult to assign to any vertebrate subtype.

The final step in apoptosis is proteolysis of a variety of target proteins in the cell by 'effector' caspases, which are activated in a proteolytic cascade by several 'apical' ('initiator') caspases [29]. Both types are clearly present in all animals (Additional data file 1). Yet, again, *Nematostella*, amphioxus, and sea urchin have representatives in more subtypes (defined by human caspases) than nematodes and insects.

## Discussion

It has been proposed that the invention of apoptosis was an essential requirement for the evolution of multicellular animals [31], and indeed it has been demonstrated that the apoptotic pathways involving members of the Bcl-2 family are present in the most basal metazoan phylum, the sponges (Porifera) [32,33]. Our results suggest that the bilaterian-cnidarian ancestor living 650-1,000 million years ago already had an apoptotic regulatory network composed of Apaf-1, Bcl-2 and caspase family members. Surprisingly, this ancient apoptosis network appears to have been more complex than previously thought and the simple networks seen in present day insects and nematodes are the result of significant gene losses. Furthermore, a central protein in the classical apoptosis model, the apoptosome forming Apaf-1 [2], which exists as a single homolog in all genomes studied so far, has multiple homologs in several morphologically simple invertebrates and many extant Apaf-1 homologs may not be orthologous. This suggests that multiple mechanisms triggering apoptosis, as well as multiple downstream pathways implementing it, may have existed in early organisms. Many gene copy number differences are found that can be explained only by lineage-specific duplications and gene losses. Apparently, different organisms evolved unique apoptosis networks, which interestingly involved essentially the same gene families, hence sometimes providing an appearance of similarity between independently evolved networks. Interestingly, apoptosis regulators are not the only protein families involved in development and disease exhibiting surprising, almost vertebrate-like complexity in Cnidaria, and thus, presumably, the common cnidarian-bilaterian ancestor [34,35]. Analyses of *Nematostella *Wnt genes revealed unforeseen ancestral diversity: *Nematostella *and bilaterians share at least eleven of the twelve known Wnt subfamilies, while five subfamilies appear to be lost in nematodes/insects [36]. Similarly, proteins with innate immunity domains have been found to be expanded in Cnidaria [37]. These results show that biological systems may not (always) evolve linearly from simple to complex. This urges caution in interpreting results from studies of *C. elegans *and *D. melanogaster *and indeed any model organisms for understanding apoptosis (or other regulatory pathways) in human. A more prudent approach might be to carefully select specific model systems for each protein family studied in such a way as to minimize the difference between the model and human. Such a selection process ideally should include phylogenetic analysis, thus reinforcing the view that "Nothing in biology makes sense except in the light of evolution." - Theodosius Dobzhansky (1900-1975).

## Conclusion

Phylogenetic inference combined with domain composition analysis of Apaf-1, Bcl-2, and caspase proteins - central players in the apoptosis network - reveal a yet unpredicted ancestral complexity within each family. In particular, the relative simplicity of these regulatory networks observed in ecdysozoan species is not the result of a gradual increase in network complexity correlating with morphological complexity, but apparently the result of widespread gene losses. Our results emphasize the importance of explicit phylogenetic analysis covering a sufficiently large sample of species space, not only in the detection of orthologous sequences, but also in model organism selection and in the study of network evolution.

## Materials and methods

### Sequence database searches

*N. vectensis *and *B. floridae *1.0 genome assemblies and protein sets were downloaded from the Joint Genome Institute [38]. The *Strongylocentrotus purpuratus *assembly Spur_v2.0 and GLEAN3 gene models were obtained from Baylor College of Medicine HGSC [39]. The other genome sequences and corresponding protein sets were downloaded from Ensembl 38 or SWISS-PROT [40,41]. Several rounds of PSI-TBLASTN searches were performed against each genome by using as seeds human NB-ARC, caspase, CARD, death, and death effector domains as well as Bcl-2 sequences from a variety of genomes [42]. The hits were then mapped to the corresponding genome protein set to acquire the full-length protein sequences (for sea urchin and *Nematostella*, some of the gene models were in addition predicted by genscan) [43]. All identified genes were checked by reciprocal BLAST analysis, Pfam 21.0 protein searches [44], Conserved Domain Search (CD-Search), and Reverse PSI-BLAST (RPS-BLAST) [45].

### Multiple sequence alignments and phylogeny reconstructions

To ensure alignment of homologous domains, sequences were trimmed to one Pfam 21.0 model (NB-ARC, Bcl-2, Peptidase_C14 for the caspase domain) [44]. Multiple sequence alignments were produced by PROBCONS 1.11 [46], MAFFT 5.861 (localpair, maxiterate 1000) [47], T-COFFEE 4.93 [48], and hmmalign from HMMER 2.3.2 [49,50]. Multiple sequence alignment columns with a gap in more than 50% of sequences were deleted. MrBayes 3.1.2 was used with 10,000,000 generations, a sample frequency of 1,000, a mixture of amino-acid models with fixed rate matrices and equal rates, and 25% burn-in [51]. For maximum likelihood approaches, PhyML 2.4.4 was used with the VT (variable time) model and four relative rate substitution categories [52,53]. Pairwise distances (for the Neighbor Joining and Fitch-Margoliash methods from PHYLIP 3.66 [54-56], and FastME 1.1 [57]) were calculated by TREE-PUZZLE 5.2 using the VT model [58]. Tree and domain composition diagrams were drawn using ATV 4a1 [59]. All conclusions presented in this work are robust relative to the alignment methods, the alignment processing, the phylogeny reconstruction methods, and the parameters used. All sequence, alignment, and phylogeny files are available upon request.

### Domain composition analysis

Domains were analyzed with hmmpfam from HMMER 2.3.2 and Pfam 21.0 [44,49], FFAS03 [60], and InterProScan [61].

## Authors' contributions

CMZ performed the phylogenetic, sequence and domain analyses of all the families in this study, as well as prepared the figures. QZ identified sequences to be analyzed and performed initial analyses. YY contributed to the domain analysis of the proteins involved in this study. AG formulated the problem and planned the work. All authors contributed to the interpretation of the results and to writing of the paper.

## Additional data files

The following additional data files are available with the online version of this paper. Additional data file 1 is a figure illustrating the evolutionary history of caspase protein family members. Additional data file 2 is a table listing the CED-4/Apaf-1 protein family members used in this study. Additional data file 3 is a table listing the multi-motif Bcl-2 protein family members used in this study. Additional data file 4 is a table listing the caspase protein family members used in this study.

## Supplementary Material

Additional data file 1 This phylogeny was calculated using a Bayesian approach (MrBayes) based on a MAFFT alignment of Peptidase_C14 domains. Posterior probability values are shown for each branch (for detailed information, see Materials and methods). Species abbreviations: BRAFL, *Branchiostoma floridae *(amphioxus); BRARE, *Brachydanio rerio *(zebrafish); CAEBR, *Caenorhabditis briggsae*; CAEEL, *Caenorhabditis elegans*; CANFA, *Canis familiaris *(dog); CHICK, *Gallus gallus *(chicken); CIOIN, *Ciona intestinalis *(sea squirt); DROME, *Drosophila melanogaster *(fruit fly); FUGRU, *Fugu rubripes *(Japanese pufferfish); NEMVE, *Nematostella vectensis *(starlet sea anemone); STRPU, *Strongylocentrotus purpuratus *(purple sea urchin); TETNG, *Tetraodon nigroviridi*s (green pufferfish); and XENTR, *Xenopus tropicalis *(western clawed frog). For a detailed list of protein sequences see Additional data file 4. Para-caspases are excluded from this phylogeny. Click here for file

Additional data file 2 Protein sequences for Figure 2 (phylogeny and domain organization of CED-4/Apaf-1 homologs). Click here for file

Additional data file 3 Protein sequences for Figure 3 (phylogeny of the multi-motif Bcl-2 family). Click here for file

Additional data file 4 Protein sequences for Additional data file 1 (phylogeny of the caspase family). Click here for file
