# Supplementary material for: Surprising complexity of the ancestral apoptosis network
Source: Genome Biol. 2007 Oct 24;8(10):R226. doi: 10.1186/gb-2007-8-10-r226 (PMC2246300; doi:10.1186/gb-2007-8-10-r226)
Supplement: Additional data file 2 — Protein sequences for Figure 2 (phylogeny and domain organization of CED-4/Apaf-1 homologs). [file gb-2007-8-10-r226-S2.pdf]

Additional Table 2

Protein sequences for Figure 2 (phylogeny and domain organization of CED-4/Apaf-1 homologs)

Abbreviation	Source and identifier
Apaf-1_HUMAN	SWISS-PROT: O14727|APAF_HUMAN
1_BRAFL	JGI Branchiostoma floridae v1.0: estExt_gwp.C_240387
2_BRAFL	JGI Branchiostoma floridae v1.0: fgenesh2_pg.scaffold_19000170
3_BRAFL	JGI Branchiostoma floridae v1.0: fgenesh2_pg.scaffold_208000046
4_BRAFL*	JGI Branchiostoma floridae v1.0: fgenesh2_pg.scaffold_265000042
5_BRAFL*	JGI Branchiostoma floridae v1.0: fgenesh2_pg.scaffold_355000025
6_BRAFL*	JGI Branchiostoma floridae v1.0: fgenesh2_pg.scaffold_460000003
7_BRAFL*	JGI Branchiostoma floridae v1.0: fgenesh2_pg.scaffold_460000017
8_BRAFL	JGI Branchiostoma floridae v1.0: fgenesh2_pg.scaffold_589000008
9_BRAFL	JGI Branchiostoma floridae v1.0: fgenesh2_pg.scaffold_879000002
CED4_CAEEL	Ensembl release_38 pep.abinitio: SNAP00000041623 U21324.3
11_CHICK	Ensembl release_38 pep.abinitio: GENSCAN00000076013 Contig36.91
12_CANFA	Ensembl release_38 pep.abinitio: GENSCAN00000000438 contig_25065.1.242843
Dark_DROME	Ensembl release_38 pep.abinitio: GENSCAN00000003874 2R_251
14_FUGRU	JGI Fugu rubripes v4.0:fgh5_pm.C_scaffold_8000017_fugu
15_TETNG	Ensembl release_38 pep.abinitio: GIDT00016877001 SCAF14664
16_XENTR	Ensembl release_38 pep.abinitio: GENSCAN00000069556 scaffold_243
17_BRARE	Ensembl release_38 pep.abinitio: GENSCAN00000025232 Zv5_NA12050.1
18_NEMVE	JGI Nematostella vectensis v1.0: scaffold_12 genscan_142
19_NEMVE	JGI Nematostella vectensis v1.0: scaffold_2 genscan_139
20_NEMVE	JGI Nematostella vectensis v1.0: scaffold_218 genscan_24
21_NEMVE	JGI Nematostella vectensis v1.0: scaffold_453 genscan_8
Apaf-1_MOUSE	SWISS-PROT: O88879|APAF_MOUSE
23_STRPU	HGSC Spur_v2.0: Scaffold_v2_43260 genscan_2
24_STRPU*	HGSC Spur_v2.0: Scaffold_v2_46046 genscan_43
25_STRPU	HGSC Spur_v2.0: Scaffold_v2_48152 genscan_19
26_STRPU	HGSC Spur_v2.0: Scaffold_v2_71267 genscan_19
27_STRPU*	HGSC Spur_v2.0: Scaffold_v2_74154 genscan_16
28_DROPS	NCBI: GI:54636004
29_AEDAE	NCBI: GI:108883840
30_TRICA	NCBI: GI:91076790
31_CAEBR	NCBI: GI:62286523
32_BRAFL*	JGI Branchiostoma floridae v1.0: fgenesh2_pg.scaffold_248000001
33_BRAFL	JGI Branchiostoma floridae v1.0: estExt_fgenesh2_pg.C_1570063
34_BRAFL	JGI Branchiostoma floridae v1.0: fgenesh2_pg.scaffold_941000003
35_BRAFL	JGI Branchiostoma floridae v1.0: fgenesh2_pg.scaffold_58000077
36_BRAFL	JGI Branchiostoma floridae v1.0: estExt_fgenesh2_pg.C_220128
37_BRAFL	JGI Branchiostoma floridae v1.0: estExt_fgenesh2_pg.C_2730005
38_BRAFL*	JGI Branchiostoma floridae v1.0: estExt_fgenesh2_pg.C_2730019

*These 8 sequences are not shown in Figure 2 because they appear redundant and/or are the result of questionable assemblies.
