# Supplementary material for: Surprising complexity of the ancestral apoptosis network
Source: Genome Biol. 2007 Oct 24;8(10):R226. doi: 10.1186/gb-2007-8-10-r226 (PMC2246300; doi:10.1186/gb-2007-8-10-r226)
Supplement: Additional data file 3 — Protein sequences for Figure 3 (phylogeny of the multi-motif Bcl-2 family). [file gb-2007-8-10-r226-S3.pdf]

Additional Table 3

Protein sequences for Figure 3 (phylogeny of the multi-motif Bcl-2 family)

Abbreviation	Source and identifier
BCL2_HUMAN	SWISS-PROT: P10415|BCL2_HUMAN
BCLX_HUMAN	SWISS-PROT: Q07817|BCLX_HUMAN
MCL1_HUMAN	SWISS-PROT: Q07820|MCL1_HUMAN
B2LA1_HUMAN	SWISS-PROT: Q16548|B2LA1_HUMAN
BAXB_HUMAN	SWISS-PROT: Q07814|BAXB_HUMAN
BAK_HUMAN	SWISS-PROT: Q16611|BAK_HUMAN
BOK_HUMAN	SWISS-PROT: Q9UMX3|BOK_HUMAN
BCLW_HUMAN	SWISS-PROT: Q92843|BCLW_HUMAN
B2L14_HUMAN	SWISS-PROT: Q9BZR8|B2L14_HUMAN
B2L10_HUMAN	SWISS-PROT: Q9HD36|B2L10_HUMAN
B2L13_HUMAN	SWISS-PROT: Q9BXK5|B2L13_HUMAN
B2L12_HUMAN	SWISS-PROT: Q9HB09|B2L12_HUMAN
BFK_HUMAN	NCBI: GI:58219054
18_MOUSE	Ensembl release_38 pep.abinitio: GENSCAN00000155005 AC131316.8
19_MOUSE	Ensembl release_38 pep.abinitio: GENSCAN00000169501 AC155838.7
20_MOUSE	Ensembl release_38 pep.abinitio: GENSCAN00000171024 AC151602.4
21_MOUSE	Ensembl release_38 pep.abinitio: GENSCAN00000175205 AL731857.9
22_MOUSE**	Ensembl release_38 pep.abinitio: GENSCAN00000175288 CAAA01175390.1
23_MOUSE*	Ensembl release_38 pep.abinitio: GENSCAN00000197433 AC156499.2
24_MOUSE*	Ensembl release_38 pep.abinitio: GENSCAN00000199518 AC126256.4
25_MOUSE	Ensembl release_38 pep.abinitio: GENSCAN00000210792 AC083894.21
26_MOUSE	Ensembl release_38 pep.abinitio: GENSCAN00000212117 AC122842.3
27_MOUSE	Ensembl release_38 pep.abinitio: GENSCAN00000212963 AC092479.16
28_MOUSE	Ensembl release_38 pep.abinitio: GENSCAN00000213484 AC124698.4
29_MOUSE	Ensembl release_38 pep.abinitio: GENSCAN00000225626 AC116591.4
30_MOUSE	Ensembl release_38 pep.abinitio: GENSCAN00000225982 AC135634.4
31_MOUSE	Ensembl release_38 pep.abinitio: GENSCAN00000195350 AC133947.3
32_CANFA	Ensembl release_38 pep.abinitio: GENSCAN00000013988 contig_47224.1.158762
33_CANFA	Ensembl release_38 pep.abinitio: GENSCAN00000014711 contig_17809.1.59485
34_CANFA*	Ensembl release_38 pep.abinitio: GENSCAN00000024591 contig_37966.1.1676
35_CANFA	Ensembl release_38 pep.abinitio: GENSCAN00000029675 contig_31336.1.156379
36_CANFA	Ensembl release_38 pep.abinitio: GENSCAN00000029721 contig_27253.1.281043
37_CANFA	Ensembl release_38 pep.abinitio: GENSCAN00000038708 contig_37968.1.11886
38_CANFA	Ensembl release_38 pep.abinitio: GENSCAN00000038911 contig_54066.1.24669
39_CANFA	Ensembl release_38 pep.abinitio: GENSCAN00000038917 contig_26592.1.2537
40_CANFA	Ensembl release_38 pep.abinitio: GENSCAN00000047139 contig_27069.1.66900
41_CANFA*	Ensembl release_38 pep.abinitio: GENSCAN00000052005 contig_27068.1.65375
42_CANFA	Ensembl release_38 pep.abinitio: GENSCAN00000057635 contig_54096.1.55228
43_CANFA	Ensembl release_38 pep.abinitio: GENSCAN00000063244 contig_17692.1.36407
44_CANFA	Ensembl release_38 pep.abinitio: GENSCAN00000069143 contig_41106.1.174558
45_CANFA	Ensembl release_38 pep.abinitio: GENSCAN00000075736 contig_14646.1.85472
46_CHICK	Ensembl release_38 pep.abinitio: GENSCAN00000001666 Contig40.185
47_CHICK	Ensembl release_38 pep.abinitio: GENSCAN00000002946 Contig4020.3
48_CHICK*	Ensembl release_38 pep.abinitio: GENSCAN00000006579 Contig4020.2
49_CHICK*	Ensembl release_38 pep.abinitio: GENSCAN00000007904 Contig40999.1
50_CHICK	Ensembl release_38 pep.abinitio: GENSCAN00000012696 Contig70.87
51_CHICK*	Ensembl release_38 pep.abinitio: GENSCAN00000018361 Contig7.471
52_CHICK*	Ensembl release_38 pep.abinitio: GENSCAN00000037013 Contig7.470
53_CHICK	Ensembl release_38 pep.abinitio: GENSCAN00000039579 Contig25.186
54_CHICK	Ensembl release_38 pep.abinitio: GENSCAN00000041103 Contig18.70
55_CHICK	Ensembl release_38 pep.abinitio: GENSCAN00000058906 Contig65.77
56_CHICK	Ensembl release_38 pep.abinitio: GENSCAN00000063752 Contig342.4
57_CHICK*	Ensembl release_38 pep.abinitio: GENSCAN00000067977 Contig65.335
58_XENTR	Ensembl release_38 pep.abinitio: GENSCAN00000016377 scaffold_587
59_XENTR	Ensembl release_38 pep.abinitio: GENSCAN00000034491 scaffold_151
60_XENTR	Ensembl release_38 pep.abinitio: GENSCAN00000039279 scaffold_1061
61_XENTR	Ensembl release_38 pep.abinitio: GENSCAN00000039870 scaffold_667
62_XENTR	Ensembl release_38 pep.abinitio: GENSCAN00000045002 scaffold_341
63_XENTR*	Ensembl release_38 pep.abinitio: GENSCAN00000045029 scaffold_341
64_XENTR	Ensembl release_38 pep.abinitio: GENSCAN00000049076 scaffold_11321
65_XENTR	Ensembl release_38 pep.abinitio: GENSCAN00000051638 scaffold_224
66_XENTR	Ensembl release_38 pep.abinitio: GENSCAN00000059978 scaffold_439
67_XENTR	Ensembl release_38 pep.abinitio: GENSCAN00000075618 scaffold_95
68_XENTR	Ensembl release_38 pep.abinitio: GENSCAN00000077815 scaffold_921
69_XENTR*	Ensembl release_38 pep.abinitio: GENSCAN00000037040 scaffold_1104

70_BRARE	Ensembl release_38 pep.abinitio: GENSCAN00000000732 Zv5_scaffold151.8
71_BRARE	Ensembl release_38 pep.abinitio: GENSCAN00000000751 AL928885.9.1-196857
72_BRARE	Ensembl release_38 pep.abinitio: GENSCAN00000016930 Zv5_scaffold1280.1
73_BRARE	Ensembl release_38 pep.abinitio: GENSCAN00000017647 AL935189.7.1-170193
74_BRARE	Ensembl release_38 pep.abinitio: GENSCAN00000017837 BX469889.7.2001-
75_BRARE	Ensembl release_38 pep.abinitio: GENSCAN00000018684 BX950194.9.1-122061
76_BRARE	Ensembl release_38 pep.abinitio: GENSCAN00000020548 BX897733.8.2001-
77_BRARE	Ensembl release_38 pep.abinitio: GENSCAN00000020578 Zv5_scaffold78.1
78_BRARE*****	Ensembl release_38 pep.abinitio: GENSCAN00000027469 Zv5_scaffold244.4
79_BRARE	Ensembl release_38 pep.abinitio: GENSCAN00000027475 Zv5_scaffold244.4
80_BRARE	Ensembl release_38 pep.abinitio: GENSCAN00000033938 Zv5_scaffold945.1
81_BRARE	Ensembl release_38 pep.abinitio: GENSCAN00000035699 BX323598.9.1-197329
82_BRARE	Ensembl release_38 pep.abinitio: GENSCAN00000036923 Zv5_scaffold1215.4
83_BRARE	Ensembl release_38 pep.abinitio: GENSCAN00000038963 Zv5_scaffold447.1
84_BRARE	Ensembl release_38 pep.abinitio: GENSCAN00000039526 BX470166.11.2001-
85_BRARE	Ensembl release_38 pep.abinitio: GENSCAN00000027319 BX248517.10.48191-
86_FUGRU	Ensembl release_38 pep.abinitio: GENSCANSLICE00000001649 scaffold_343
87_FUGRU	Ensembl release_38 pep.abinitio: GENSCANSLICE00000004269 scaffold_73
88_FUGRU	Ensembl release_38 pep.abinitio: GENSCANSLICE00000005941 scaffold_285
89_FUGRU	Ensembl release_38 pep.abinitio: GENSCANSLICE00000012751 scaffold_41
90_FUGRU	Ensembl release_38 pep.abinitio: GENSCANSLICE00000014016 scaffold_79
91_FUGRU*****	Ensembl release_38 pep.abinitio: GENSCANSLICE00000015032 scaffold_85
92_FUGRU	Ensembl release_38 pep.abinitio: GENSCANSLICE00000015496 scaffold_41
93_FUGRU	Ensembl release_38 pep.abinitio: GENSCANSLICE00000020201 scaffold_175
94_FUGRU	Ensembl release_38 pep.abinitio: GENSCANSLICE00000021934 scaffold_370
95_FUGRU	Ensembl release_38 pep.abinitio: GENSCANSLICE00000022364 scaffold_174
96_FUGRU*****	Ensembl release_38 pep.abinitio: GENSCANSLICE00000022457 scaffold_67
97_FUGRU	Ensembl release_38 pep.abinitio: GENSCANSLICE00000028825 scaffold_65
98_FUGRU	Ensembl release_38 pep.abinitio: GENSCANSLICE00000028826 scaffold_65
99_FUGRU	Ensembl release_38 pep.abinitio: GENSCANSLICE00000026604 scaffold_128
100_TETNG	Ensembl release_38 pep.abinitio: GIDT00016701001 SCAF14653
101_TETNG	Ensembl release_38 pep.abinitio: GSCT00000143001 SCAF14528
102_TETNG	Ensembl release_38 pep.abinitio: GSCT00001457001 SCAF14566
103_TETNG	Ensembl release_38 pep.abinitio: GSCT00003389001 SCAF14729
104_TETNG	Ensembl release_38 pep.abinitio: GSCT00005369001 SCAF14992
105_TETNG	Ensembl release_38 pep.abinitio: GSCT00005404001 SCAF14992
106_TETNG	Ensembl release_38 pep.abinitio: GSCT00011096001 SCAF7631
107_TETNG	Ensembl release_38 pep.abinitio: GSCT00016806001 SCAF3587
108_TETNG	Ensembl release_38 pep.abinitio: GSCT00018946001 SCAF10464
109_TETNG	Ensembl release_38 pep.abinitio: GSCT00022958001 SCAF13692
110_TETNG	Ensembl release_38 pep.abinitio: GWSST00007956001 SCAF14700
111_TETNG*****	Ensembl release_38 pep.abinitio: GWSST00017694001 SCAF3333
112_TETNG******	Ensembl release_38 pep.abinitio: GWSST00017797001 SCAF3587
113_BRAFL	JGI Brachiostoma floridae v1.0: e_gw.30.321.1
114_BRAFL	JGI Brachiostoma floridae v1.0: e_gw.394.70.1
115_BRAFL	JGI Brachiostoma floridae v1.0: e_gw.493.26.1
116_BRAFL	JGI Brachiostoma floridae v1.0: fgenesh2_pg.scaffold_16000109
117_BRAFL	JGI Brachiostoma floridae v1.0: fgenesh2_pg.scaffold_2229000001
118_BRAFL	JGI Brachiostoma floridae v1.0: fgenesh2_pg.scaffold_73000155
119_BRAFL	JGI Brachiostoma floridae v1.0: fgenesh2_pg.scaffold_91000061
120_CIOIN	Ensembl release_38 pep.abinitio: GENSCAN00000094137 4q_317
121_STRPU	HGSC Spur_v2: Scaffold_v2_39347_genscan_25 Scaffold_v2_39347
122_STRPU	HGSC Spur_v2: Scaffold_v2_86134_genscan_4 Scaffold_v2_86134
123_STRPU	HGSC Spur_v2: Scaffold_v2_44505_genscan_12 Scaffold_v2_44505
124_STRPU	HGSC Spur_v2: Scaffold_v2_86172_genscan_1 Scaffold_v2_86172
125_STRPU	HGSC Spur_v2: Scaffold_v2_5975_genscan_1 Scaffold_v2_5975
126_STRPU	HGSC Spur_v2: Scaffold_v2_33343_genscan_2 Scaffold_v2_33343
127_STRPU	HGSC Spur_v2: Scaffold_v2_65149_genscan_12 Scaffold_v2_65149
128_STRPU	HGSC Spur_v2: Scaffold_v2_9776_genscan_3 Scaffold_v2_9776
Debcl_DROME	Ensembl release_38 pep.abinitio: GENSCAN00000014809 2R_44
Buffy_DROME	Ensembl release_38 pep.abinitio: SNAP00000038892 2R_145
CED9_CAEEL	Ensembl release_38 pep.abinitio: GENEFINDER00000031967 Z29443.1
CED9_CAEBR	SWISS-PROT: P41957|CED9_CAEBR
136_NEMVE	JGI Nematostella vectensis v1.0: e_gw.125.48.1
137_NEMVE	JGI Nematostella vectensis v1.0: e_gw.255.21.1
138_NEMVE	JGI Nematostella vectensis v1.0: e_gw.472.19.1
139_NEMVE	JGI Nematostella vectensis v1.0: e_gw.55.77.1
140_NEMVE	JGI Nematostella vectensis v1.0: e_gw.56.102.1
141_NEMVE	JGI Nematostella vectensis v1.0: estExt_fgenesh1_pg.C_1400041
142_NEMVE	JGI Nematostella vectensis v1.0: estExt_fgenesh1_pg.C_2300022
143_NEMVE	JGI Nematostella vectensis v1.0: fgenesh1_pg.scaffold_1000191
144_GEOCY	EMBL: AJ293508
145_LUBBA	NCBI: GI:92011891

146_SUBDO	NCBI: GI:55770952
147_HYDAT	NCBI: GI:118505072
281_MOUSE	NCBI: GI:2493276
282_MOUSE	NCBI: GI:21553305

*These 15 sequences are not shown in Figure 3 because they match the Bcl-2 ls HMM (Pfam 21.0) with an E-value larger than 0.3.

**These 2 sequences are not shown in Figure 3 because they appear redundant and/or are the result of questionable assemblies.
