# Supplementary material for: Surprising complexity of the ancestral apoptosis network
Source: Genome Biol. 2007 Oct 24;8(10):R226. doi: 10.1186/gb-2007-8-10-r226 (PMC2246300; doi:10.1186/gb-2007-8-10-r226)
Supplement: Additional data file 4 — Protein sequences for Additional data file 1 (phylogeny of the caspase family). [file gb-2007-8-10-r226-S4.pdf]

Additional Table 4

Protein sequences for Additional Figure 4 (phylogeny of the caspase family)

**Abbreviation**	**Source and identifier**
CASP1_HUMAN	SWISS-PROT: P29466|CASP1_HUMAN
CASP2_HUMAN	SWISS-PROT: P42575|CASP2_HUMAN
CASP3_HUMAN	SWISS-PROT: P42574|CASP3_HUMAN
CASP4_HUMAN	SWISS-PROT: P49662|CASP4_HUMAN
CASP5_HUMAN	SWISS-PROT: P51878|CASP5_HUMAN
CASP6_HUMAN	SWISS-PROT: P55212|CASP6_HUMAN
CASP7_HUMAN	SWISS-PROT: P55210|CASP7_HUMAN
CASP8_HUMAN	SWISS-PROT: Q14790|CASP8_HUMAN
CASP9_HUMAN	SWISS-PROT: P55211|CASP9_HUMAN
CASP10_HUMAN	SWISS-PROT: Q92851|CASPA_HUMAN
CASP14_HUMAN	SWISS-PROT: P31944|CASPE_HUMAN
MALT1_HUMAN **p**	SWISS-PROT: Q9UDY8|MALT1_HUMAN
CFLAR_HUMAN	SWISS-PROT: O15519|CFLAR_HUMAN
CASP12_HUMAN	SWISS-PROT: Q6UXS9|Q6UXS9_HUMAN
14_BRAFL	JGI Branchiostoma floridae v1.0: e_gw.281.109.1
15_BRAFL	JGI Branchiostoma floridae v1.0: e_gw.289.46.1
16_BRAFL	JGI Branchiostoma floridae v1.0: e_gw.317.50.1
17_BRAFL	JGI Branchiostoma floridae v1.0: e_gw.323.60.1
18_BRAFL	JGI Branchiostoma floridae v1.0: e_gw.38.32.1
19_BRAFL	JGI Branchiostoma floridae v1.0: e_gw.41.74.1
20_BRAFL	JGI Branchiostoma floridae v1.0: e_gw.440.22.1
21_BRAFL	JGI Branchiostoma floridae v1.0: e_gw.457.27.1
22_BRAFL	JGI Branchiostoma floridae v1.0: e_gw.558.40.1
23_BRAFL	JGI Branchiostoma floridae v1.0: e_gw.58.117.1
24_BRAFL	JGI Branchiostoma floridae v1.0: e_gw.58.143.1
25_BRAFL	JGI Branchiostoma floridae v1.0: e_gw.624.12.1
26_BRAFL	JGI Branchiostoma floridae v1.0: e_gw.752.1.1
27_BRAFL	JGI Branchiostoma floridae v1.0: e_gw.79.62.1
28_BRAFL	JGI Branchiostoma floridae v1.0: e_gw.87.121.1
29_BRAFL	JGI Branchiostoma floridae v1.0: estExt_fgenesh2_pg.C_1570004
30_BRAFL	JGI Branchiostoma floridae v1.0: estExt_fgenesh2_pg.C_570070
31_BRAFL	JGI Branchiostoma floridae v1.0: estExt_gwp.C_380044
32_BRAFL	JGI Branchiostoma floridae v1.0: estExt_gwp.C_5310019
33_BRAFL	JGI Branchiostoma floridae v1.0: fgenesh2_kg.scaffold_76000001
34_BRAFL	JGI Branchiostoma floridae v1.0: fgenesh2_pg.scaffold_106000101
35_BRAFL	JGI Branchiostoma floridae v1.0: fgenesh2_pg.scaffold_11000166
36_BRAFL	JGI Branchiostoma floridae v1.0: fgenesh2_pg.scaffold_114000007
37_BRAFL	JGI Branchiostoma floridae v1.0: fgenesh2_pg.scaffold_116000092
38_BRAFL	JGI Branchiostoma floridae v1.0: fgenesh2_pg.scaffold_12000073
39_BRAFL	JGI Branchiostoma floridae v1.0: fgenesh2_pg.scaffold_128000077
40_BRAFL	JGI Branchiostoma floridae v1.0: fgenesh2_pg.scaffold_14000081
41_BRAFL	JGI Branchiostoma floridae v1.0: fgenesh2_pg.scaffold_14000189
42_BRAFL	JGI Branchiostoma floridae v1.0: fgenesh2_pg.scaffold_163000014
43_BRAFL	JGI Branchiostoma floridae v1.0: fgenesh2_pg.scaffold_17000032
44_BRAFL	JGI Branchiostoma floridae v1.0: fgenesh2_pg.scaffold_179000014
45_BRAFL	JGI Branchiostoma floridae v1.0: fgenesh2_pg.scaffold_189000062
46_BRAFL	JGI Branchiostoma floridae v1.0: fgenesh2_pg.scaffold_206000012
47_BRAFL	JGI Branchiostoma floridae v1.0: fgenesh2_pg.scaffold_224000042
48_BRAFL	JGI Branchiostoma floridae v1.0: fgenesh2_pg.scaffold_257000041
49_BRAFL	JGI Branchiostoma floridae v1.0: fgenesh2_pg.scaffold_266000032
50_BRAFL	JGI Branchiostoma floridae v1.0: fgenesh2_pg.scaffold_440000013
51_BRAFL	JGI Branchiostoma floridae v1.0: fgenesh2_pg.scaffold_44000123
52_BRAFL	JGI Branchiostoma floridae v1.0: fgenesh2_pg.scaffold_505000011
53_BRAFL	JGI Branchiostoma floridae v1.0: fgenesh2_pg.scaffold_505000014
54_BRAFL	JGI Branchiostoma floridae v1.0: fgenesh2_pg.scaffold_55000086
55_BRAFL	JGI Branchiostoma floridae v1.0: fgenesh2_pg.scaffold_58000144
56_BRAFL	JGI Branchiostoma floridae v1.0: fgenesh2_pg.scaffold_85000001
57_BRAFL	JGI Branchiostoma floridae v1.0: fgenesh2_pg.scaffold_96000010
58_BRAFL	JGI Branchiostoma floridae v1.0: gw.24.194.1 scaffold_24
59_BRAFL	JGI Branchiostoma floridae v1.0: gw.3.308.1 scaffold_3
60_BRAFL	JGI Branchiostoma floridae v1.0: gw.41.75.1 scaffold_41
61_BRAFL	JGI Branchiostoma floridae v1.0: gw.457.36.1 scaffold_457
62_BRAFL	JGI Branchiostoma floridae v1.0: gw.5.697.1 scaffold_5
63_BRAFL	JGI Branchiostoma floridae v1.0: gw.527.30.1 scaffold_527
64_BRAFL	JGI Branchiostoma floridae v1.0: gw.531.4.1 scaffold_531

65_BRAFL*****	JGI Branchiostoma floridae v1.0: gw.752.6.1 scaffold_752
66_BRAFL	JGI Branchiostoma floridae v1.0: gw.97.227.1 scaffold_97
67_CAEEL **p**	Ensembl release_38 pep.abinitio: SNAP00000012440 U28993.2
CED3_CAEEL	Ensembl release_38 pep.abinitio: SNAP00000017192 Z81049.1
CSP3_CAEEL	Ensembl release_38 pep.abinitio: SNAP00000020500 AL032657.1
CSP2_CAEEL	Ensembl release_38 pep.abinitio: SNAP00000037670 AC084197.1
CSP1_CAEEL	Ensembl release_38 pep.abinitio: SNAP00000039230 Z93393.2
72_CHICK	Ensembl release_38 pep.abinitio: GENSCAN00000017364 Contig150.9
73_CHICK	Ensembl release_38 pep.abinitio: GENSCAN00000028400 Contig19.506
74_CHICK	Ensembl release_38 pep.abinitio: GENSCAN00000031849 Contig22.130
75_CHICK	Ensembl release_38 pep.abinitio: GENSCAN00000037777 Contig3.1036
76_CHICK	Ensembl release_38 pep.abinitio: GENSCAN00000038708 Contig75.50
77_CHICK	Ensembl release_38 pep.abinitio: GENSCAN00000038723 Contig75.50
78_CHICK	Ensembl release_38 pep.abinitio: GENSCAN00000038726 Contig75.50
79_CHICK	Ensembl release_38 pep.abinitio: GENSCAN00000038728 Contig75.50
80_CHICK **p**	Ensembl release_38 pep.abinitio: GENSCAN00000043145 Contig106.83
81_CHICK	Ensembl release_38 pep.abinitio: GENSCAN00000062140 Contig3.422
82_CHICK*****	Ensembl release_38 pep.abinitio: GENSCAN00000064390 Contig184.95
83_CHICK*****	Ensembl release_38 pep.abinitio: GENSCAN00000070643 Contig184.94
84_CHICK	Ensembl release_38 pep.abinitio: GENSCAN00000071772 Contig1601.9
85_CIOIN	Ensembl release_38 pep.abinitio: FGENESH00000070934 12q_24
86_CIOIN	Ensembl release_38 pep.abinitio: GENEFINDER00000089975 12q_245
87_CIOIN	Ensembl release_38 pep.abinitio: GENSCAN00000077382 12p_145
88_CIOIN	Ensembl release_38 pep.abinitio: GENSCAN00000078600 8q_205
89_CIOIN	Ensembl release_38 pep.abinitio: GENSCAN00000080393 12q_223
90_CIOIN	Ensembl release_38 pep.abinitio: GENSCAN00000080400 12q_219
91_CIOIN	Ensembl release_38 pep.abinitio: GENSCAN00000088212 scaffold_180_7
92_CIOIN	Ensembl release_38 pep.abinitio: GENSCAN00000091551 8q_418
93_CIOIN*****	Ensembl release_38 pep.abinitio: GENSCAN00000091653 6q_75
94_CIOIN	Ensembl release_38 pep.abinitio: GENSCAN00000102076 4q_272
95_CIOIN	Ensembl release_38 pep.abinitio: SNAP_CIONA00000044087 12q_220
110_CANFA	Ensembl release_38 pep.abinitio: GENSCAN00000003002 contig_48336.1.38164
111_CANFA **p**	Ensembl release_38 pep.abinitio: GENSCAN00000020890 contig_14601.1.99562
112_CANFA	Ensembl release_38 pep.abinitio: GENSCAN00000022553 contig_16054.1.355264
113_CANFA	Ensembl release_38 pep.abinitio: GENSCAN00000027274 contig_38046.1.61723
114_CANFA	Ensembl release_38 pep.abinitio: GENSCAN00000030806 contig_44577.1.10789
115_CANFA	Ensembl release_38 pep.abinitio: GENSCAN00000044240 contig_45483.1.101664
116_CANFA	Ensembl release_38 pep.abinitio: GENSCAN00000044314 contig_23073.1.2928
117_CANFA	Ensembl release_38 pep.abinitio: GENSCAN00000049145 contig_54810.1.318000
118_CANFA	Ensembl release_38 pep.abinitio: GENSCAN00000049146 contig_54810.1.318000
119_CANFA	Ensembl release_38 pep.abinitio: GENSCAN00000049148 contig_54810.1.318000
120_CANFA	Ensembl release_38 pep.abinitio: GENSCAN00000071074 contig_21074.1.14608
121_CANFA	Ensembl release_38 pep.abinitio: GENSCAN00000075637 contig_55271.1.46482
122_CANFA	Ensembl release_38 pep.abinitio: GENSCAN00000077085 contig_9193.1.230116
123_CANFA	Ensembl release_38 pep.abinitio: GENSCAN00000077086 contig_9193.1.230116
DAMM_DROME	Ensembl release_38 pep.abinitio: GENSCAN00000016500 2R_148
Strica_DROME	Ensembl release_38 pep.abinitio: GENSCAN00000005714 2R_31
Dcp1_DROME	Ensembl release_38 pep.abinitio: GENSCAN00000010154 2R_382
Dronc_DROME	Ensembl release_38 pep.abinitio: GENSCAN00000003674 3L_199
Decay_DROME	Ensembl release_38 pep.abinitio: GENSCAN00000003643 3R_247
drICE_DROME	Ensembl release_38 pep.abinitio: GENSCAN00000014490 3R_513
DREDD_DROME	Ensembl release_38 pep.abinitio: GENSCAN00000016383 X_10
131_FUGRU	Ensembl release_38 pep.abinitio: GENSCANSLICE00000008704 scaffold_466
132_FUGRU*****	Ensembl release_38 pep.abinitio: GENSCANSLICE00000010825 scaffold_46
133_FUGRU	Ensembl release_38 pep.abinitio: GENSCANSLICE00000011518 scaffold_4498
134_FUGRU	Ensembl release_38 pep.abinitio: GENSCANSLICE00000012037 scaffold_17
135_FUGRU	Ensembl release_38 pep.abinitio: GENSCANSLICE00000015664 scaffold_100
136_FUGRU **p**	Ensembl release_38 pep.abinitio: GENSCANSLICE00000017185 scaffold_4
137_FUGRU **p**	Ensembl release_38 pep.abinitio: GENSCANSLICE00000019238 scaffold_135
138_FUGRU	Ensembl release_38 pep.abinitio: GENSCANSLICE00000020903 scaffold_263
139_FUGRU **p**	Ensembl release_38 pep.abinitio: GENSCANSLICE00000021754 scaffold_217
140_FUGRU	Ensembl release_38 pep.abinitio: GENSCANSLICE00000026027 scaffold_387
141_FUGRU	Ensembl release_38 pep.abinitio: GENSCANSLICE00000028088 scaffold_9
142_FUGRU	Ensembl release_38 pep.abinitio: GENSCANSLICE00000028892 scaffold_81
143_FUGRU	Ensembl release_38 pep.abinitio: GENSCANSLICE00000029213 scaffold_17
144_MOUSE	Ensembl release_38 pep.abinitio: GENSCAN00000154690 AC112968.11
145_MOUSE	Ensembl release_38 pep.abinitio: GENSCAN00000156226 AC115771.13
146_MOUSE	Ensembl release_38 pep.abinitio: GENSCAN00000169177 AL928577.21
147_MOUSE	Ensembl release_38 pep.abinitio: GENSCAN00000170756 AC119267.8
148_MOUSE*****	Ensembl release_38 pep.abinitio: GENSCAN00000181320 CAAA01088007.1
149_MOUSE	Ensembl release_38 pep.abinitio: GENSCAN00000191566 AC141637.3
150_MOUSE	Ensembl release_38 pep.abinitio: GENSCAN00000196011 AC159747.8
151_MOUSE	Ensembl release_38 pep.abinitio: GENSCAN00000211658 AC118620.10

152_MOUSE **p**	Ensembl release_38 pep.abinitio: GENSCAN00000218568 AC102240.12
153_TETNG	Ensembl release_38 pep.abinitio: GIDT00013441001 SCAF14542
154_TETNG*****	Ensembl release_38 pep.abinitio: GIDT00022814001 SCAF15004
155_TETNG	Ensembl release_38 pep.abinitio: GSCT00000593001 SCAF14542
156_TETNG	Ensembl release_38 pep.abinitio: GSCT00000759001 SCAF14543
157_TETNG	Ensembl release_38 pep.abinitio: GSCT00002741001 SCAF14659
158_TETNG **p**	Ensembl release_38 pep.abinitio: GSCT00005619001 SCAF14995
159_TETNG **p**	Ensembl release_38 pep.abinitio: GSCT00005723001 SCAF14996
160_TETNG*****	Ensembl release_38 pep.abinitio: GSCT00006606001 SCAF15004
161_TETNG	Ensembl release_38 pep.abinitio: GSCT00007603001 SCAF15038
162_TETNG **p**	Ensembl release_38 pep.abinitio: GSCT00008343001 SCAF15122
163_TETNG	Ensembl release_38 pep.abinitio: GSCT00009329001 SCAF15010
164_TETNG*****	Ensembl release_38 pep.abinitio: GSCT00011521001 SCAF14157
165_TETNG	Ensembl release_38 pep.abinitio: GWSST00003280001 SCAF14485
166_TETNG	Ensembl release_38 pep.abinitio: GWSST00015337001 SCAF15106
167_XENTR	Ensembl release_38 pep.abinitio: FGENESH00000087096 scaffold_90
168_XENTR **p**	Ensembl release_38 pep.abinitio: GENSCAN00000011636 scaffold_103
169_XENTR	Ensembl release_38 pep.abinitio: GENSCAN00000013067 scaffold_89
170_XENTR	Ensembl release_38 pep.abinitio: GENSCAN00000034007 scaffold_736
171_XENTR **p**	Ensembl release_38 pep.abinitio: GENSCAN00000037477 scaffold_228
172_XENTR	Ensembl release_38 pep.abinitio: GENSCAN00000038004 scaffold_90
173_XENTR	Ensembl release_38 pep.abinitio: GENSCAN00000039972 scaffold_135
174_XENTR	Ensembl release_38 pep.abinitio: GENSCAN00000055657 scaffold_218
175_XENTR	Ensembl release_38 pep.abinitio: GENSCAN00000055664 scaffold_218
176_XENTR	Ensembl release_38 pep.abinitio: GENSCAN00000055667 scaffold_218
177_XENTR	Ensembl release_38 pep.abinitio: GENSCAN00000066282 scaffold_1113
178_XENTR	Ensembl release_38 pep.abinitio: GENSCAN00000068240 scaffold_30
179_XENTR	Ensembl release_38 pep.abinitio: GENSCAN00000074022 scaffold_1474
180_BRARE*****	Ensembl release_38 pep.abinitio: FGENESH00000074106 Zv5_NA4522.1
181_BRARE	Ensembl release_38 pep.abinitio: GENSCAN00000002628 Zv5_scaffold449.1
182_BRARE	Ensembl release_38 pep.abinitio: GENSCAN00000004209 Zv5_scaffold1510.1
183_BRARE	Ensembl release_38 pep.abinitio: GENSCAN00000004413 BX005348.9.1-145299
184_BRARE	Ensembl release_38 pep.abinitio: GENSCAN00000004425 BX323548.11.1-229237
185_BRARE	Ensembl release_38 pep.abinitio: GENSCAN00000010360 BX649388.7.1-177865
186_BRARE	Ensembl release_38 pep.abinitio: GENSCAN00000010681 BX537280.12.1-163151
187_BRARE **p**	Ensembl release_38 pep.abinitio: GENSCAN00000013724 AL935332.12.2001-
188_BRARE	Ensembl release_38 pep.abinitio: GENSCAN00000020009 Zv5_scaffold816.8
189_BRARE	Ensembl release_38 pep.abinitio: GENSCAN00000024545 Zv5_NA4522.1
190_BRARE	Ensembl release_38 pep.abinitio: GENSCAN00000024624 Zv5_scaffold1161.3
191_BRARE	Ensembl release_38 pep.abinitio: GENSCAN00000027488 Zv5_scaffold66.3
192_BRARE	Ensembl release_38 pep.abinitio: GENSCAN00000028136 Zv5_scaffold1016.17
193_BRARE **p**	Ensembl release_38 pep.abinitio: GENSCAN00000029654 Zv5_scaffold1233.1
194_BRARE	Ensembl release_38 pep.abinitio: GENSCAN00000031927 BX005142.4.2001-
195_BRARE **p**	Ensembl release_38 pep.abinitio: GENSCAN00000034621 BX323550.13.1-200031
196_BRARE **p**	Ensembl release_38 pep.abinitio: GENSCAN00000034633 Zv5_scaffold1096.18
197_BRARE	Ensembl release_38 pep.abinitio: GENSCAN00000035392 BX469930.13.2001-
198_BRARE	Ensembl release_38 pep.abinitio: GENSCAN00000036728 AL929213.5.1-169897
199_BRARE	Ensembl release_38 pep.abinitio: GENSCAN00000037707 Zv5_scaffold340.4
200_BRARE	Ensembl release_38 pep.abinitio: GENSCAN00000042057 Zv5_scaffold819.2
201_NEMVE	JGI Nematostella vectensis v1.0: e_gw.224.21.1
202_NEMVE	JGI Nematostella vectensis v1.0: e_gw.360.15.1
203_NEMVE	JGI Nematostella vectensis v1.0: e_gw.384.21.1
204_NEMVE	JGI Nematostella vectensis v1.0: e_gw.57.87.1
205_NEMVE	JGI Nematostella vectensis v1.0: e_gw.98.208.1
206_NEMVE	JGI Nematostella vectensis v1.0: estExt_fgenesh1_pm.C_660002
207_NEMVE	JGI Nematostella vectensis v1.0: estExt_fgenesh1_pm.C_740006
208_NEMVE	JGI Nematostella vectensis v1.0: estExt_gwp.C_4180040
209_NEMVE **p**	JGI Nematostella vectensis v1.0: fgenesh1_pg.scaffold_140000023 scaffold_140
210_NEMVE	JGI Nematostella vectensis v1.0: gw.158.70.1
211_STRPU*****	HGSC Spur_v2.0: Scaffold_v2_102470_genscan_1
212_STRPU	HGSC Spur_v2.0: Scaffold_v2_10454_genscan_2
213_STRPU	HGSC Spur_v2.0: Scaffold_v2_11300_genscan_7
214_STRPU	HGSC Spur_v2.0: Scaffold_v2_11320_genscan_2
215_STRPU	HGSC Spur_v2.0: Scaffold_v2_17921_genscan_1
216_STRPU	HGSC Spur_v2.0: Scaffold_v2_22981_genscan_2
217_STRPU	HGSC Spur_v2.0: Scaffold_v2_22981_genscan_4
218_STRPU	HGSC Spur_v2.0: Scaffold_v2_22981_genscan_10
219_STRPU	HGSC Spur_v2.0: Scaffold_v2_22981_genscan_12
220_STRPU*****	HGSC Spur_v2.0: Scaffold_v2_23814_genscan_1
221_STRPU	HGSC Spur_v2.0: Scaffold_v2_23814_genscan_2
222_STRPU	HGSC Spur_v2.0: Scaffold_v2_2419_genscan_1
223_STRPU	HGSC Spur_v2.0: Scaffold_v2_27169_genscan_1
224_STRPU	HGSC Spur_v2.0: Scaffold_v2_28078_genscan_24

225_STRPU	HGSC Spur_v2.0: Scaffold_v2_28078_genscan_27
226_STRPU	HGSC Spur_v2.0: Scaffold_v2_28115_genscan_1
227_STRPU	HGSC Spur_v2.0: Scaffold_v2_29819_genscan_1
228_STRPU*****	HGSC Spur_v2.0: Scaffold_v2_3438_genscan_6
229_STRPU	HGSC Spur_v2.0: Scaffold_v2_35424_genscan_9
230_STRPU	HGSC Spur_v2.0: Scaffold_v2_38293_genscan_1
231_STRPU	HGSC Spur_v2.0: Scaffold_v2_4297_genscan_1
232_STRPU	HGSC Spur_v2.0: Scaffold_v2_4297_genscan_3
233_STRPU	HGSC Spur_v2.0: Scaffold_v2_43352_genscan_2
234_STRPU	HGSC Spur_v2.0: Scaffold_v2_4343_genscan_3
235_STRPU	HGSC Spur_v2.0: Scaffold_v2_4343_genscan_4
236_STRPU*****	HGSC Spur_v2.0: Scaffold_v2_4979_genscan_4
237_STRPU	HGSC Spur_v2.0: Scaffold_v2_50167_genscan_2
238_STRPU	HGSC Spur_v2.0: Scaffold_v2_50936_genscan_1
239_STRPU	HGSC Spur_v2.0: Scaffold_v2_58909_genscan_1
240_STRPU	HGSC Spur_v2.0: Scaffold_v2_59540_genscan_13
241_STRPU	HGSC Spur_v2.0: Scaffold_v2_61322_genscan_2
242_STRPU	HGSC Spur_v2.0: Scaffold_v2_6185_genscan_1
243_STRPU	HGSC Spur_v2.0: Scaffold_v2_63743_genscan_1
244_STRPU **p**	HGSC Spur_v2.0: Scaffold_v2_65983_genscan_1
245_STRPU	HGSC Spur_v2.0: Scaffold_v2_68537_genscan_1
246_STRPU*****	HGSC Spur_v2.0: Scaffold_v2_69977_genscan_1
247_STRPU	HGSC Spur_v2.0: Scaffold_v2_70603_genscan_1
248_STRPU*****	HGSC Spur_v2.0: Scaffold_v2_83186_genscan_19
249_STRPU	HGSC Spur_v2.0: Scaffold_v2_83903_genscan_16
250_STRPU	HGSC Spur_v2.0: Scaffold_v2_86595_genscan_2
251_STRPU*****	HGSC Spur_v2.0: Scaffold_v2_86851_genscan_1
252_STRPU*****	HGSC Spur_v2.0: Scaffold_v2_96440_genscan_1

*****These 18 sequences are not shown in shown in Additional Figure 4 because they match the Peptidase_C14 ls HMM (Pfam 21.0) with an E-value larger than 0.1 and/or because the match length is less than 100aa.

p: These (19) para-caspase sequences are not shown in shown in Additional Figure 4.
